# Differences in the impedance of cochlear implant devices within 24 hours of their implantation

**DOI:** 10.1371/journal.pone.0222711

**Published:** 2019-09-19

**Authors:** David Po-Yi Lin, Joshua Kuang-Chao Chen, Tao-Hsin Tung, Lieber Po-Hung Li

**Affiliations:** 1 Department of Otolaryngology, Cheng Hsin General Hospital, Taipei, Taiwan; 2 Cochlear Implant Center, Far Eastern Memorial Hospital, New Taipei, Taiwan; 3 Department of Medical Research and Education, Cheng Hsin General Hospital, Taipei, Taiwan; 4 Faculty of Medicine, School of Medicine, National Yang-Ming University, Taipei, Taiwan; 5 Institute of Brain Science, School of Medicine, National Yang-Ming University, Taipei, Taiwan; Harvard Medical School Teaching Hospital, UNITED STATES

## Abstract

Cochlear implantation is a surgical procedure, which is performed on severely hearing-impaired patients. Impedance field telemetry is commonly used to determine the integrity of the cochlear implant device during and after surgery. At the Department of Otolaryngology, Cheng Hsin General Hospital (Taipei, Taiwan), the cochlear implant devices are switched on within 24 hours of their implantation. In the present study, the impedance changes of Advanced Bionics^™^ cochlear implant devices were compared with previous studies and other devices. The aim was to confirm previous hypotheses and to explore other potential associated factors that could influence impedance following cochlear implantation. The current study included 12 patients who underwent cochlear implantation at Cheng Hsin General Hospital with Advanced Bionics cochlear implant devices. The cochlear devices were all switched on within 24 hours of their implantation. The impedance was measured and compared across all contact channels of the electrode, both intra-operatively and post-operatively. The intra-operative impedance was compared with the switch-on impedance (within 24 hours of the cochlear implantation); the impedance was notably increased for all contact channels at switch-on. Of the 16 channels examined, 4 channels had a significant increase in impedance between the intra-operative measurement and the switch-on measurement. To the best of our knowledge, the impedance of a cochlear implant device can be affected by the diameter of the electrode, the position of the electrode arrays in the scala tympani, sheath formation and fibrosis surrounding the electrode after implantation and electrical stimulation during or after surgery. When the results of the current study were compared with previous studies, it was found that the impedance changes were opposite to that of Cochlear^™^ implant devices. This may be explained by the position of the electrode arrays, sheath formation, the blow-out effect and differences in electrical stimulation.

## Introduction

Cochlear implants are devices, which are implanted into the cochlea to aid hearing via stimulation of the cochlear nerve. The incidence of cochlear implantation has increased throughout the world over the past few decades, and it is considered one of the best medical interventions for patients with severe to profound sensorineural hearing loss. Over the last few years, Cheng Hsin General Hospital have routinely performed successful initial cochlear implant switch-on within 24 hours of their implantation [1–5].

Impedance field telemetry is one of the most commonly used parameters to assess the integrity of the device during implantation and mapping. In a previous study by the authors, a significant drop in impedance was found during initial mapping within 24 hours of the cochlear implantation [1]. This finding may have been associated with the spontaneous recovery of the microenvironment inside the cochlea and a divergence effect of electrical stimulation after the device was switched on.

In the present study, the impedance changes in patients implanted with Advanced Bionics cochlear implants were measured within 24 hours of the surgery being performed. The results were then compared with previous studies and it was revealed that the results were inconsistent. Previous studies have discussed the possible effects of tissue fibrosis, micro-environmental changes and electrical stimulation on impedance changes, however none have specifically investigated Advanced Bionics cochlear implants with initial switch-on within 24 hours of their implantation or made comparisons with other implant devices. To the best of our knowledge, the current study is the first to discuss impedance changes on different cochlear implant devices within 24 hours of their implantation into humans. The aim of the study was to confirm previous hypotheses such as the size of the electrode, fibrosis formation and electrical stimulation. We also wish to explore other associated factors that may influence impedance changes after cochlear implantation.

## Materials

### Patients

A total of 12 patients who received Advanced Bionics HiFocus1J cochlear implant systems at Cheng Hsin General Hospital between May 2007 and June 2015, and underwent initial switch-on within 24 hours of the operation, were included in the present study. Of the 12 included patients, 7 received cochlear implants on the right side and 5 received them on the left. The Institutional Ethics and Research Committee of Cheng Hsin General Hospital approved the current retrospective review and the requirement for informed consent was waived.

### Surgery

The surgical techniques used were the same for all patients. They included 2.5–3 cm post-auricular incision wounds, drilling of a bone housing for the receiver-stimulator, harvesting of a cortex bone chip from the mastoid using a minimal mastoidectomy, a posterior tympanostomy, hyaluronic acid gel coverage of the cochleostomy prior to array insertion, utilization of the soft insertion technique and sheltering of any defects of the mastoid cavity with the harvested bone chip after insertion. Full insertion of the electrode array was confirmed in each patient by a postoperative X-ray [1–3]. All speech processors were switched on within 24 hours of the operation. All intra-operative impedance measurements were acquired right after the implantation in the operation room, and all post-operative measurements were acquired after cochlear implant activation the next day. The electrode configuration for all cochlear implant devices in this study were monopolar. Impedance measurements were performed using SoundWave^™^ version 3.1 provided by Advanced Bionics.

### Statistical analysis

All statistical analyses were performed using SPSS version 18.0.0 (SPSS, Inc., Chicago, IL, USA). A paired sample t-test was used to compare values from consecutive fitting sessions. Continuous data were presented as the mean ± standard deviation (SD). Statistical significance was set as P < 0.05.

## Results

All patients successfully underwent cochlear implant activation within 24 hours of the device implantation. They each had a small incision wound of around 2.5–3 cm and there were no complications, such as post-operative infection, hematoma or pain noted by any patient.

**Fig 1:**
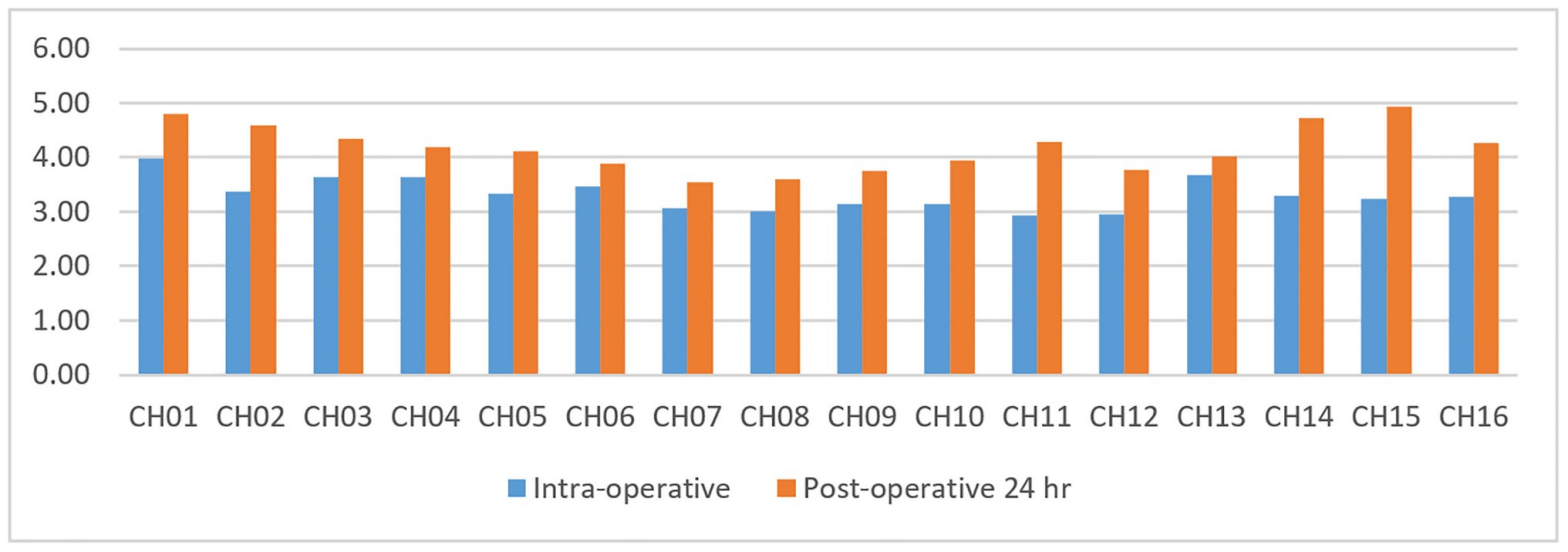
Intra-operative and 24 hour post-operative impedance measurement averages. CH: channel.

The impedance measured within 24 hours of the device insertion was notably increased on all electrode contact channels, compared with the impedance measured intra-operatively (Fig 1). This increase was significant for CH 2 (P = 0.030), CH 11 (P = 0.033), CH 15 (P = 0.023) and CH 16 (P = 0.049) (Table 1). In addition, no short or open circuits of the devices were noted intra- or post-operatively.

**Table 1:** Comparison of intra-operative and 24 hour post-operative impedance measurements.

	Intra-operative -Average	Intra-operative -SD	Post-operative 24 hour -Average	Post-operative 24 hour -SD	P-value of Averages
CH01	3.98	2.04	4.79	2.43	0.065
CH02	3.38	0.98	4.58	2.22	0.030*
CH03	3.64	1.29	4.35	1.96	0.120
CH04	3.63	1.47	4.19	1.94	0.162
CH05	3.33	1.49	4.12	2.11	0.111
CH06	3.48	1.75	3.89	2.10	0.200
CH07	3.08	1.45	3.54	2.15	0.191
CH08	3.00	1.24	3.59	2.03	0.134
CH09	3.14	1.44	3.76	2.18	0.095
CH10	3.14	1.55	3.94	2.41	0.065
CH11	2.93	1.65	4.29	3.62	0.033*
CH12	2.95	1.12	3.78	2.36	0.091
CH13	3.68	1.75	4.03	2.25	0.326
CH14	3.30	1.83	4.73	3.17	0.068
CH15	3.23	1.91	4.93	3.47	0.023*
CH16	3.28	2.19	4.26	3.49	0.049*

Impedance unit: kOhm. CH: channel. SD: standard deviation.

*P < 0.05.

## Discussion

The current study identified a significant rise in impedance at 24 hours after surgery, in patients who received Advanced Bionics HiFocus1J cochlear implants. This finding was different from a previous study by the authors, which investigated patients with Cochlear^™^ implants, in which a significant drop in the level of impedance was observed within 24 hours of their implantation [1, 3]. The findings of the current study were also inconsistent with a previous study on animals, which claimed that post-implantation electrical detection levels were significantly decreased at several days after surgery [6]. To the best of our knowledge, the present study is the first to discuss the impedance changes of different cochlear implant devices within 24 hours of their implantation in humans. Although the pathophysiological and micro-environment changes after cochlear implantation are still not fully understood, some associated factors that may affect the impedance level after implantation have been previously discussed. These include, the diameter of the electrode, sheath formation and fibrosis surrounding the electrode after implantation and electrical stimulation during or after surgery.

### Sheath formation and fibrosis surrounding the electrode after implantation

One possible factor that could affect the impedance of cochlear implants is sheath formation and fibrosis around the electrodes [7, 8]. Previous studies have hypothesized that a tissue sheath forms around the electrodes shortly after their insertion [9, 10]. This sheath could then cause an increase in the impedance of the electrode contact channels, which are dependent on the interaction between the degree of cell cover formation and on site fibrosis [11, 12]. Previous studies have reported that cell cover formation may be influenced by the diameter of the electrode [12]. In a previous study by the authors, patients were implanted with Cochlear Contour Advance electrodes, which have electrode arrays of 0.5 mm in diameter over the apex and 0.8 mm over the base. In the current study, the patients were implanted with Advanced Bionics Hi Focus1J electrodes, which have electrode arrays of 0.4 mm in diameter over the apex and 0.8 mm over the base. There is very little difference in the diameter of the electrodes between these two studies, which makes it hard to explain the drastic differences in impedance changes using array diameters. However, the position of the arrays in the scala tympani differed. Due to its pre-curved design, a Cochlear Contour Advance electrode sits much closer to the modiolus than an Advanced Bionics Hi Focus1J electrode, which may give the latter more room to develop sheath formation and fibrosis. This could result in a more significant rise in impedance after implantation[13, 14].

### Electrical stimulation during or after the implantation

Another hypothesis for impedance changes is the blow-out effect. This starts with the reorganization of the thin and newly-formed tissue sheath or the formation of bubbles around the electrodes, which are caused by electrical stimulation from the initial switch-on. These changes in the status of the tissue sheath and bubble formation around the electrodes may result in a decrease in impedance. It can be assumed that the strength of this electrical stimulation is equal to the power dissipating through the electrodes. According to Ohm’s law, electrical power varies directly with the electrical resistance and the square of the electrical current. The Advanced Bionics implants are given an electrical current up to a maximum of 300 mA, while the Cochlear implants are given an electrical current up to a maximum of 250–300 mA. However, the electrical resistance is 2.1–3.9 kilohms for the Advanced Bionics implants, and 5.1–11.7 kilohms for the Cochlear implants. This results in a higher level of power dissipating through the electrodes of the Cochlear implants, which causes a drop in impedance within 24 hours after implantation. However, this was not observed in the current study where Advanced Bionics cochlear implants were used.

In addition, Advanced Bionics^™^ implants are not pre-conditioned, meaning that no electrical current passed through the electrodes during phase 1 prior to impedance measurements whereas Cochlear implants are. This supports the hypothesis that the impedance was affected by the blow-out effect, causing a drop before its measurement for the Cochlear implants compared with the Advanced Bionics implants.

### Limitations

The current study used a relatively small sample size. Future studies should include a larger number of subjects to confirm the proposed theories.

## Conclusions

The current study revealed that impedance changes were inconsistent among different studies and cochlear implant devices. Instead of dropping as in previous studies, the impedance in the present study rose within 24 hours after cochlear implantation. Previous studies have discussed the possible effects of tissue fibrosis, micro-environment changes and electrical stimulation on impedance, however none have specifically studied Advanced Bionics cochlear implants with an initial switch-on within 24 hours of their implantation or compared them with different devices. To the best of our knowledge, the current study is the first to discuss impedance changes on different cochlear implant devices within 24 hours of their implantation in humans.

There is still much to learn about the pathophysiological and micro-environmental changes that occur after cochlear implantation. However, using the available data, the most probable explanation for the difference in impedance between the Advanced Bionics and Cochlear implants are the position of the electrode arrays in the scala tympani and the varying electrical stimulus patterns of the different devices. The results from this study could potentially have a great impact on the choice of cochlear implant activation timing, device selection and electrical stimulation patterns in the future.

## Supporting information

S1 File Raw data of impedance measurements. (XLSX)
